# Effects of Gahvora Cradling on Learning, Motor Behavior, and Expression of Affect in 3-Month-Olds in Tajikistan

**DOI:** 10.3390/brainsci16070736

**Published:** 2026-07-12

**Authors:** Lana B. Karasik, Scott R. Robinson, Rano Dodojonova

**Affiliations:** 1 Department of Psychology, College of Staten Island & Graduate Center, City University of New York, 2800 Victory Blvd., 4S-220, Staten Island, NY 10314, USA; 2 Pacific Ethological Laboratories, Olympia, WA 98512, USA; 3 Scientific-Clinical Center for Pediatrics & Children’s Surgery, 59 I. Somoni Str., Building 7, Floor 3, Dushanbe 734026, Tajikistan

**Keywords:** motor development, cross-cultural differences, perceptual–motor development

## Abstract

**Highlights:**

**What are the main findings?**

- Three-month-old infants reared in restrictive gahvora cradles for most of each day demonstrated robust contingency learning in a mobile paradigm: they selectively increased kicking of the operative leg, differentiated it from the untethered leg, and retained the learned association during extinction—the hallmarks of contingency detection.
- Time out of the gahvora shaped how learning unfolded but not whether it occurred: infants tested immediately after release started with lower baseline kicking yet converged with peers by extinction, and negative affect rose specifically when the contingency was removed, indicating that all infants formed expectations about the action–outcome relation.

**What are the implications of the main findings?**

- Contingency detection emerges rapidly whenever infants have the opportunity for self-produced action, indicating a sensorimotor learning mechanism driven by available movement affordances and operating on a fast timescale.
- Cultural childrearing practices that extensively restrict infant movement shape the circumstances of early contingent experience without limiting infants’ fundamental capacity to learn from those experiences—a finding that may help explain the absence of lasting developmental lags in gahvora-reared children and refines theories linking motor experience to early cognitive development.

**Abstract:**

Background/Objectives: We examined spontaneous movement in 26 3-month-olds in Tajikistan, all reared in a “gahvora” cradle from birth for most of each day. The gahvora swaddles and binds infants’ limbs and torso, restricting movement. Methods: Infants participated in a conjugate reinforcement mobile paradigm immediately after release from the gahvora or after several minutes of unrestricted activity. Infants completed the task at home across five 3 min blocks: baseline, three acquisition blocks, and extinction. Coders scored tethered and untethered leg kicks from video in every block and also scored facial expressions and vocalizations to track affect. Mothers reported typical gahvora use. Results: All infants learned: kicks of the tethered leg exceeded kicks of the untethered leg, and kicking remained elevated at extinction relative to baseline. Learning rates diverged most between infants tested immediately after release from the gahvora and infants who had several minutes of unrestricted activity beforehand. Conclusions: A within-culture comparison of infants’ exposure to body and limb restriction reveals how cultural childrearing practices shape opportunities for perceptual–motor development.

## 1. Effects of Gahvora Cradling on Learning, Motor Behavior, and Expression of Affect in 3-Month-Olds in Tajikistan

### 1.1. Contingency Learning in Early Development

Sensitivity to contingencies anchors early cognitive and social development. Even in the first months after birth, infants demonstrate an acute awareness of how their actions influence physical objects and social partners. They quickly discern that batting a mobile sets it moving, that pressing a button produces sound, and that crying summons caregivers. During face-to-face interaction, mothers echo babies’ vocalizations, and infants, recognizing the response, vocalize again, creating a back-and-forth social exchange. Through everyday experiences, infants learn that their behaviors have meaningful consequences that lay an early foundation for goal-directed behavior. Learning depends on infants’ self-generated actions—their spontaneous movements of eyes, limbs, body, facial expressions, and vocalizations. Without self-initiated behaviors, infants would lack the input needed to detect and learn from contingent associations among their actions and the resulting consequences. Sensitivity to self-generated action–outcome associations does not emerge at birth; fetal behavioral work shows that contingency detection begins prenatally. This is implied by adaptive motor responses to experimental occlusion of the umbilical cord [1] and experimentally confirmed by explicit training of interlimb coordination [2]. The result is that infants arrive primed to register how their actions produce consequences [3,4].

Motor development provides the fundamental basis for these early learning experiences. As infants gain increasing control over their limbs and bodies, they discover that their specific movements yield predictable outcomes. Modern theories of motor development emphasize that infants must move freely and explore how their movements relate to environmental features [5,6]. One of the earliest paradigms demonstrating this visual–motor contingency learning is mobile conjugate reinforcement [7,8]. In this paradigm, an infant’s leg connects to an overhead mobile via a ribbon, such that kicking produces corresponding movement of the mobile. Within minutes, infants as young as 3 months increase their kicking rate by 2–3 times baseline after discovering the contingency. They also retain the learned association when tested days later [9], suggesting that contingency detection shapes immediate behavior and also contributes to early memory systems. In North American samples, 80–90% of 3-month-olds show learning within a single session. The robust learning shows how strongly infants seek to detect and exploit causal relations in their environment.

Cradling practices in Tajikistan, Central Asia, have infants in extended motor restriction during the months when contingency learning takes place. Infants spend 18–22 h per day at 3 months of age in a gahvora cradle that binds the limbs, torso, and head [10]. The cradle includes a built-in drainage system allowing infants to remain clean and dry for hours without unwrapping. The degree and duration of gahvora use is in stark contrast to the shorter-term swaddling described in Western infant-care research, which is reserved only for sleep and practiced only for the first three months of life [10,11]. When removed from the gahvora, infants typically experience periods of caregiving activities including feeding, bathing, and social interaction, during which they have freedom of movement.

In non-human animals, sustained immobilization in early development depresses motor behavior for hours to days after release [12,13]. Prior studies documented similar effects in human infants whose movement or posture was restricted by childrearing practices like swaddling, cradleboard use, and the traditional Chinese practice of sandbagging [14,15,16,17]. The magnitude of these effects varies with the degree and duration of restriction. Swaddling reduced infants’ arousal; swaddled infants slept more, startled less, and showed a lower and less variable heart rate, suggesting that swaddling reduced arousal through reduced self-generated movement [18]. Infants who slept in the supine position—a posture-based restriction rather than global immobilization—reached several early milestones later than prone sleepers, though all infants eventually performed within accepted developmental ranges [15]. Ethnological studies of Navajo cradleboard use found no detectable effects on motor milestones because infants spent most restricted time asleep and time on the cradleboard declined sharply within the first year [14]. Infants reared in sand-filled bags for more than 16 h per day showed delays in sitting and walking [16]. The gahvora cradle used in Tajikistan represents a more extreme form of restriction than any of those described above: full-body swaddling while supine and sustained for many hours per day throughout the first two years of life. Moreover, this caregiving practice is virtually universal in Tajikistan today. Given this intensity and duration, the gahvora would seem to produce substantial and lasting developmental differences. Yet our earlier work found only transient effects—modest variation in sitting and walking that converged with World Health Organization standards [19]—showing that infants acquired all typical motor skills despite extended physical restriction.

Orphanage-reared infants reveal what makes the gahvora context different. Orphanage-reared infants in Hungary showed pronounced delays across motor, cognitive, and linguistic domains [20]. Researchers often have attributed these delays to limited movement and social interaction, but the delays may also stem from a lack of response-contingent experience. That is, orphanage-reared infants may lack both physical opportunities for self-generated movement and consistently responsive caregivers. Gahvora-reared infants, by contrast, have access to both freedom of movement and responsive caregivers [10].

Two possible explanations for the transient effects of motor restriction in Tajik infants include responsive social interaction during out-of-cradle periods and sensorimotor recalibration when movement becomes possible. Because gahvora-reared infants share a broadly similar restriction history, naturally occurring variation in how recently infants were last out of the cradle offers a window on the recalibration mechanism, isolating the contribution of recent movement opportunity and holding restriction history roughly constant. Infants released from the gahvora may have a motor system still accustomed to restraint and may need a period of readjustment to the unrestricted environment, similar to the recalibration rat pups and human infants demonstrate during experimentally manipulated environments [21,22], or after the prenatal-to-postnatal transition, when fetuses move from a uterine context to a markedly different sensorimotor environment [23,24].

### 1.2. Current Study

Childrearing practices in Tajikistan involve a degree of restriction that contrasts sharply with typical North American and European practices. To investigate how restricted movement affects contingency learning, we recruited Tajik infants to participate in the mobile conjugate reinforcement paradigm, examining three interrelated outcomes: contingency learning, motor activity levels, and expression of affect. Examining these three dimensions allows us to distinguish whether any observed patterns in contingency learning reflect genuine differences in learning capacity, altered behavioral state, or emotional reactivity to the novel testing situation. The emotion, motor, and cognitive systems are deeply interconnected across early development, such that changes in one system reverberate through the others [25,26]. Infants’ motor experiences, emotional responses, and attentional and appraisal processes co-develop as a functionally integrated system rather than as independent capacities that come online separately [27].

We asked whether 3-month-old gahvora-reared infants would show contingency learning in the mobile paradigm. Three theoretical scenarios were possible. Because fetuses respond to their own movements and to tactile and proprioceptive feedback in utero [23,24], complete absence of contingency detection in gahvora-reared infants was unlikely. We therefore considered how learning might be reduced or slowed in its expression. First, if contingency learning requires extensive prior experience with self-generated movement and its consequences, gahvora-reared infants might show reduced learning compared to Western samples, who do not typically experience extensive motor restriction and have more opportunities for free movement. Such a pattern would suggest that the quantity of movement experience during early development is critical for establishing basic contingency detection. Second, if out-of-cradle experiences provide sufficient input for developing sensitivity to action–outcome relations, gahvora-reared infants might show learning comparable to infants with unrestricted movement. Such a pattern would indicate that limited movement in one domain (motor) does not preclude changes in another (cognitive) when contingent experiences are available. Third, even if infants lack extensive movement history, they might rapidly discover and exploit contingencies when opportunities arise. Under the third scenario, gahvora-reared infants at 3 months—when gahvora use is high—would show learning but with slower initial acquisition as they adapt in real time to the sudden freedom of movement during testing.

Because all infants experience periodic removal from the gahvora for caregiving, they have some exposure to spontaneous movement, but their response in the mobile paradigm would reflect immediate adaptation to unrestricted limb movement rather than drawing on an established repertoire of action–outcome knowledge. Such a pattern would suggest that contingency detection is robust and rapidly available, but its expression depends on current motor affordances rather than accumulated movement experience. Three-month-olds offer a window onto the sensorimotor loop. By 3 months, infants with unrestricted movement couple their limb actions to environmental consequences [8,28]. How that coupling forms under severe restriction reveals whether the loop builds from accumulated practice or comes online the moment contingent action becomes possible.

Beyond learning ability, we examined two additional factors that could affect outcomes in this mobile training setting. First, we examined baseline kicking rate before the contingency was introduced as an index of motor activity and arousal independent of contingency learning. Previous research shows that swaddling reduces distress and keeps infants calm even after swaddling is removed [29,30,31], and swaddling improves attention, with greater previous swaddling experience seeming to increase attention spans [32]. The gahvora, however, differs from swaddling as described in the literature: The duration and degree of restraint in the gahvora far exceed anything studied in Western swaddling research, where infants are typically swaddled for short stretches and retain some range of limb movement. Gahvora-reared infants might therefore show either reduced baseline kicking due to habituation to restraint and enhanced behavioral regulation, or increased baseline kicking as a rebound response to sudden freedom of movement. We examined baseline kicking rate as an indicator of behavioral state and arousal that could affect their ability to detect and respond to the contingency. Measuring baseline kicking rates is important because low rates might preclude sufficient response during the training trials and high rates might prevent infants showing any increase in kicking over trials.

Second, we assessed negative affect in the mobile conjugate reinforcement paradigm because arousal is thought to modulate contingency learning [33,34]. Infants who enter the mobile task in a state of heightened emotional reactivity, whether distressed or excitedly aroused, may attend less to the leg-to-mobile relation, whereas infants who are calm and engaged may more readily pick up on action–outcome contingencies. Motor restriction that has just been removed could plausibly push arousal in either direction, and so we tracked affect together with kicking to separate contingency detection from its affective behaviors. Studies of infant temperament show that some infants respond to an overhead mobile with enhanced reactivity, whereas others show less reactivity [35,36]. High-reactive infants respond to mobiles—especially those with many moving parts—with increased motor activity and signs of negative affect. If Tajik infants’ emotional state after release from the gahvora results in an abundance of sensory stimulation, both from the infants’ new freedom of movement and the novelty of a mobile, then changes in affective reactivity might account for measured responses to contingent reinforcement.

The three theoretical scenarios outlined above suggested three different patterns of infant response to mobile training: (1) they might demonstrate little or no learning, (2) they might immediately respond to mobile training with no period of adaptation after restriction, or (3) they might need some time to adjust after removal from the gahvora to effectively respond to training. We hypothesized that gahvora-reared infants would demonstrate contingency learning, albeit potentially with an initial delay or attenuated response magnitude compared to unrestricted Western samples reported in prior work because infants already possess a fundamental learning mechanism that can be activated rapidly when environmental conditions permit self-generated action. Second, we hypothesized that negative affect would not account for individual differences in contingency learning, indicating that the novel testing situation does not produce excessive distress in this population.

## 2. Methods

### 2.1. Participants

The Tajik co-author, R.D., recruited families from medical clinics based on infants’ age, term birth, and no known birth complications. The researcher informed medical personnel and mothers that the purpose of the study was to learn about infants’ daily routines and development. Verbal consent was obtained from parents prior to participation; the University Integrated Institutional Review Board of the City University of New York (IRB #440783-CSI) approved the study and the verbal consent procedures; approval has been continuous since 2013, with the most recent renewal in 2025. The study was conducted in accordance with the Declaration of Helsinki. Families consented to their infant’s participation prior to any data collection. Families received toys and other baby products for participation.

The final sample consisted of *N* = 26 mothers and their 3-month-old (±1 week) infants (15 girls and 11 boys). An additional 7 infants were excluded from analyses due to: equipment failure (*n* = 1), excessive fussiness preventing task completion (*n* = 5), and experimenter error (*n* = 1).

The mothers’ ages ranged from 19 to 36 years (*M* = 26.90 years, *SD* = 2.83), and they had between 1 and 6 children at the time of data collection (*M* = 2.33 children). All mothers breastfed infants from birth. Most mothers (63%) had completed secondary school (11 years of education), 22% had finished primary school (4 years), 7% had no education, and 7% had completed more than 11 years of schooling. Most mothers did not work for pay (87%), 8.5% worked on a collective farm, and the rest (5.1%) did odd jobs. All mothers were married. Fathers (49%) were migrant workers in Russia and did not live at home, 26% lived at home and did odd jobs, 13% worked in construction, 5% were drivers, and 7% did not work. All mothers spoke Tajik as their primary language; none spoke English. Sessions were conducted in Tajik by a native Tajik speaker.

To characterize infants’ movement restriction history, mothers reported on their infant’s gahvora use during the previous 24 h period, particularly whether infants spent time in it during the day and night. Mothers confirmed that infants started using gahvoras shortly after birth (*M* = 9.38 days old, *SD* = 9.97) and spent days and nights in gahvoras.

### 2.2. Procedure, Materials, and Apparatus

The Tajik co-author, R.D., visited families at home for approximately 1 h at a convenient time for caregivers. Visits included (1) interviews with caregivers about gahvora use and (2) observations of infants in the conjugate mobile task. The mobile task began after the infant was released from the gahvora during the home visit; the interval between release and task onset was not experimentally standardized and instead reflected family scheduling and the researcher’s arrival time. We describe this variation when defining the delay-based subgroup analysis below.

**Figure 1:**
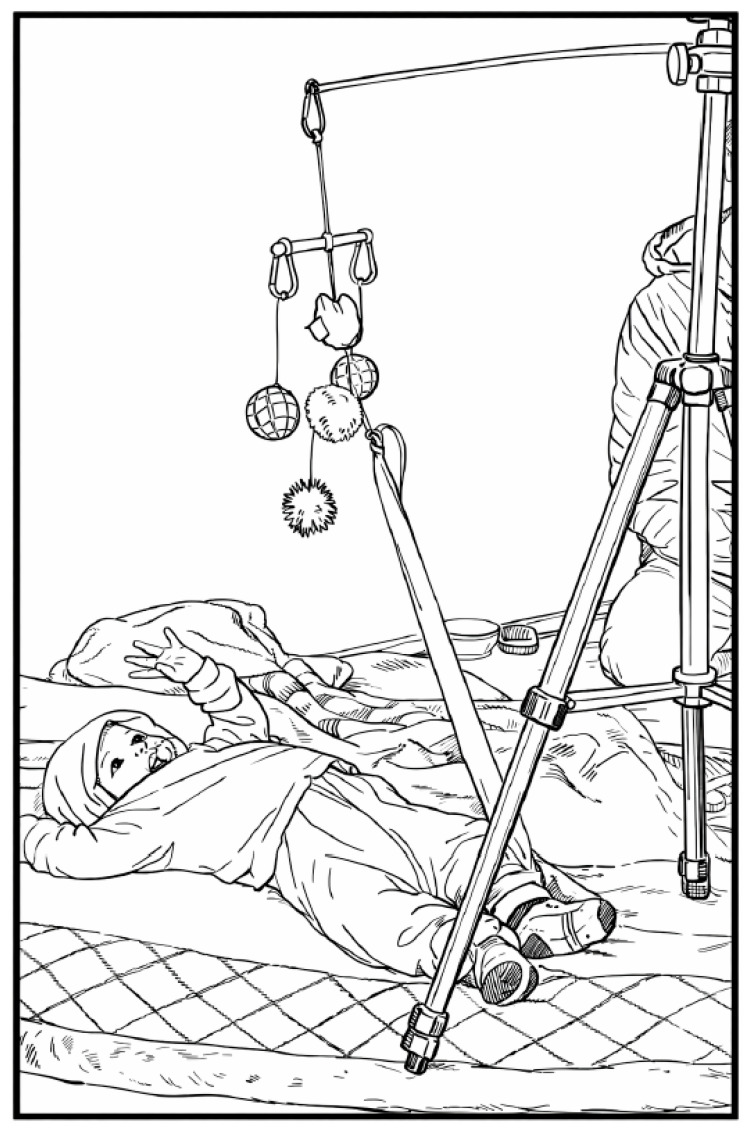
Mobile task. Illustration of the mobile conjugate reinforcement paradigm as custom-built for Tajik infants. The infant lies supine on the floor with a ribbon connecting one ankle to an overhead mobile. Leg kicks activate the mobile, allowing the infant to discover the contingent relation between their actions and the mobile’s movement.

The mobile conjugate reinforcement paradigm followed procedures established by Rovee-Collier and colleagues [8,37,38,39]. Infants were placed supine on a blanket on the floor in an area of the home. We built a custom mobile apparatus (Figure 1), which was suspended approximately 50–60 cm above the infants’ chest, positioned within the infant’s visual field. The mobile consisted of 5 colorful objects attached to a wooden rod crossbar (approximately 30 cm in length): one blue foam butterfly (approximately 8 cm), two hollow plastic balls with openings (one hot pink, one orange, each approximately 6 cm diameter), one lime green fuzzy pom-pom (approximately 5 cm diameter), and one smaller blue spiky ball (approximately 4 cm diameter). The objects were suspended at varying heights from the crossbar using thin string, creating movement at different levels. The crossbar was attached to a tripod stand via carabiners and string, allowing the entire mobile to move when activated. A soft fabric ribbon (approximately 2.5 cm wide, hot pink) was attached to the infant’s ankle and connected to the mobile apparatus via the crossbar attachment point, such that leg kicks produced visible vertical and rotational movement of the mobile objects and crossbar.

The task consisted of five 3 min trial blocks across three phases: one baseline, three acquisition blocks, and one extinction. During baseline, the ribbon was attached to the infant’s ankle but not connected to the mobile, providing a measure of spontaneous kicking in the absence of contingency. During the three blocks of acquisition, the ribbon was connected to the mobile so that kicks produced corresponding mobile movement. During the extinction block, the ribbon was again disconnected from the mobile to assess whether kicking rates remained elevated after contingency removal. A caregiver remained near the infant throughout the procedure but was instructed not to interact with the infant unless the infant became distressed. If the infant became excessively fussy or upset, the session was discontinued.

### 2.3. Data Coding and Reliability

Video records were coded using Datavyu (datavyu.org), a computerized video coding system which allows frame-by-frame coding of frequencies and durations of specific behaviors time-locked to videos.

The primary coder identified each leg kick during all three phases, baseline, acquisition, and extinction. A kick was defined as any extension or upward thrust of the leg of sufficient force to displace the mobile. During contingent (acquisition) phases, coders established an amplitude and force threshold by requiring that a kick produce an audible ring of the bell attached to the mobile; the coder used that ring-generating threshold as a proxy for kick force in the baseline and extinction phases, which were coded after the contingent phases. The ribbon itself was not used to define a kick. The acquisition ratio, calculated as the kicking rate during the final acquisition block divided by the kicking rate during the baseline block, with ratios ≥ 1.5 indicating learning.

The primary coder identified each vocalization produced during all three phases. She marked the start of a vocalization when infants began to produce a sound and marked the offset when the vocalization ended. She then classified the vocalization as negative if infants produced a whimper, fussing, or crying sound accompanied by a corresponding *negative* facial expression (furrowed eyebrows, downward mouth). Canonical or pre-canonical vocalizations were classified as *positive/neutral*. Ambiguous vocalizations were classified as negative if accompanied by a negative facial expression (furrowed eyebrows and downward mouth).

Reliability. A second coder independently coded 25% of all participants’ sessions to assess inter-rater reliability. Intraclass correlation coefficients (ICCs) for continuous measures were kicking ICC = 0.97 and negative affect ICC = 0.95. For categorical judgments of affect, Cohen’s kappa = 0.84.

## 3. Results

We examined whether 3-month-old gahvora-reared infants demonstrated contingency learning in the mobile conjugate reinforcement paradigm and whether learning was influenced by time out of the gahvora. We analyzed three primary outcomes: (1) evidence of contingency learning as measured by changes in kicking across blocks; (2) the effect of time out of the gahvora on baseline motor activity and subsequent learning; and (3) patterns of vocalization and affect as infants learned and experienced removal of the contingency. Data were analyzed using repeated-measures and univariate analyses of variance (ANOVAs), with follow-up planned comparisons and correlational analyses where appropriate. Preliminary analyses revealed no differences between boys and girls on any measure, so subsequent analyses collapsed across infant sex, all *p*s > 0.05.

### 3.1. Evidence of Contingency Learning

**Figure 2:**
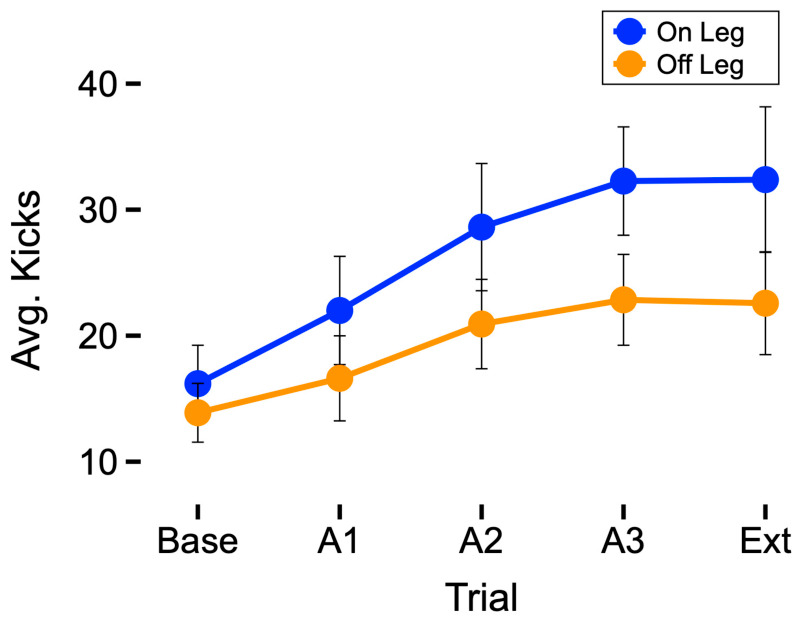
Kicking rate of tethered (“on”) and untethered (“off”) legs across five test blocks. Kicking increases over the three acquisition blocks relative to baseline and is more pronounced in the on-leg than the off-leg. Kicking rates remain elevated during the final extinction block.

Our primary question asked whether 3-month-old gahvora-reared infants would demonstrate contingency learning in the mobile paradigm. We conducted a 2 (leg: tethered vs. untethered) × 5 (blocks: baseline, acquisition 1–3, extinction) repeated-measures ANOVA on kicking frequency. As shown in Figure 2, infants showed evidence of learning the contingency between their kicks and mobile movement.

We found a main effect of trial block, *F*(4, 100) = 5.31, *p* < 0.001. Infants increased their kicking beginning in the second acquisition block. Relative to baseline, infants kicked more frequently during acquisition blocks 2 and 3, and kicking remained elevated during the extinction block, suggesting retention of the learned association even after the contingency was removed.

Following convention in mobile conjugate reinforcement research, we classified infants as learners if their kicking rate reached at least 1.5 times their baseline rate in the last acquisition block [28]. Out of the 26 infants, 14 (53.8%) met this learning criterion. Beyond categorical classification, we examined the strength of learning using the acquisition ratio (mean kicking rate during the final acquisition block divided by baseline kicking rate). An acquisition ratio greater than 1.5 indicates that kicking increased above baseline, with higher ratios reflecting stronger learning. Two infants had zero kicks at baseline, yielding an undefined acquisition ratio, and were excluded from ratio analyses. Across the remaining 24 infants, the mean acquisition ratio was *M* = 3.92 (*SD* = 6.27, range = 0–25.00), indicating that learning occurred at the group level, *t*(23) = 1.89, *p* < 0.05, *d* = 0.63. The Cohen’s *d* falls in the medium range, suggesting a reasonably robust learning effect at the group level but wide individual differences; some infants were strong learners and others learned barely at all.

Infants differentiated between the two legs during training, the tethered leg compared to the untethered leg, *F*(1, 25) = 5.31, *p* < 0.05. We found an interaction between leg and block *F*(4, 100) = 3.46, *p* < 0.05, indicating that differences between the two legs were most pronounced during acquisition blocks 3 and 4 and during extinction. No differences between the two legs were found at baseline or during the first acquisition, indicating that leg differentiation emerged as infants learned the contingency rather than reflecting pre-existing motor asymmetries.

### 3.2. The Role of Time out of Gahvora

**Figure 3:**
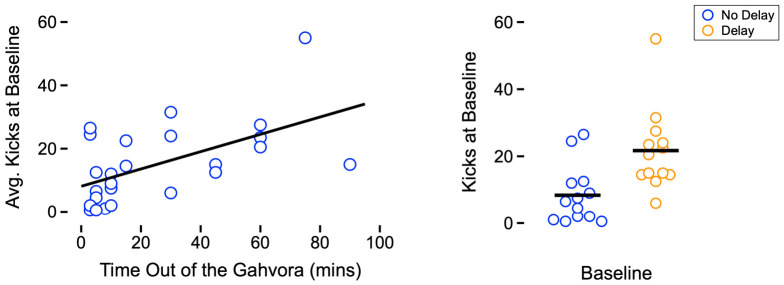
Baseline kicking as a function of time out of the gahvora and delay group assignment. The left panel shows a scatterplot of average kicks at baseline as a function of time out of the gahvora (in minutes) prior to the start of the session for all infants. The trend line indicates a positive association between time out of the gahvora and baseline kicking. The right panel shows individual baseline kick rates for infants in the no-delay group (blue circles) and the delay group (orange circles). Infants in the delay group, who had been out of the gahvora longer before testing began, showed higher baseline kicking than infants in the no-delay group. Horizontal lines indicate group means.

A critical question was whether the observed learning pattern reflected genuine contingency detection or simply increasing motor activity as infants gradually “warmed up” after release from the gahvora. We noticed substantial individual variation in baseline kicking that appeared to be related to how long infants had been out of the gahvora before beginning the task. Time out of the gahvora ranged from 3 to 90 min (*M* = 25.40 min, *SD* = 25.98). Average kicking at baseline (averaged across both legs) ranged from 0.5 to 55 kicks (*M* = 15.04, *SD* = 12.37). Figure 3 (left panel) shows the positive correlation between kicking and time out of the gahvora, *r*(24) = 0.56, *p* < 0.01, indicating that infants who had been out of the gahvora longer kicked more frequently at baseline.

To examine whether this baseline variability affected our findings, we divided the sample using a 10 min criterion (see Figure 3, right panel), creating two groups: the “delay” group (*n* = 13), infants who had at least 10 min out of the gahvora before beginning the mobile task, and the “no-delay” group (*n* = 13), infants who began the task within 10 min of gahvora removal.

### 3.3. Contingency Learning Across Delay Groups

**Figure 4:**
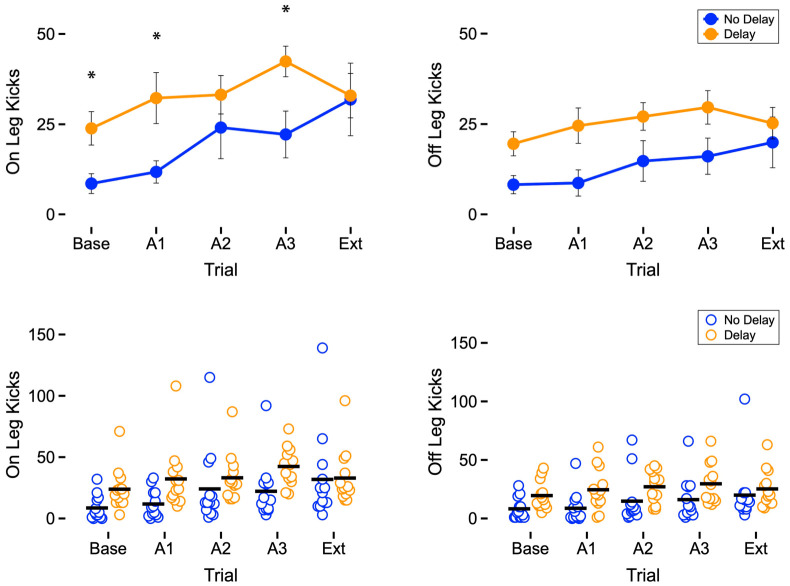
Leg kicks across acquisition and extinction blocks for the no-delay (blue) and delay (orange) groups. The asterisk indicates differences (*p* < 0.05) between the two groups. The left panels show kicks of the tethered (on) leg, and the right panels show kicks of the untethered (off) leg. Top panels display group means with standard error bars; bottom panels display individual data points with group means indicated by horizontal lines. Both groups increased kicking of the tethered leg across acquisition blocks, with the delay group showing consistently higher kick rates. Kicks of the untethered leg showed a smaller block effect than the tethered leg, and neither the group nor interaction terms reached significance.

Figure 4 shows the frequency of kicking for the tethered leg (left panels) and untethered leg (right panels) across blocks for both delay groups. Time out of the gahvora ranged continuously from 15 to 90 min; the primary test of its effect on tethered-leg kicking modeled time as a continuous predictor, and delay-group comparisons serve as a categorical, visualization-friendly secondary view and as the only available test for the untethered leg. We conducted two GLM analyses—separate 2 (delay group) × 5 (block) mixed-design ANOVAs—to model the effects of group and block on kicking frequency for the tethered and untethered legs.

For the tethered leg, there was a main effect of block, *F*(4, 96) = 7.14, *p* < 0.001, partial η^2^ = 0.23. Notably, there was also an interaction between delay group and block, *F*(4, 96) = 2.43, *p* = 0.05, partial η^2^ = 0.10. Because the kicking distributions were positively skewed and overdispersed relative to a Poisson assumption (Shapiro–Wilk *p* < 0.001 at four of five blocks), we confirmed this interaction with a negative binomial GEE modeling kicks as a function of block, group, and their interaction; the block × group interaction was robust, Wald χ^2^(4) = 25.28, *p* < 0.001. Planned comparisons revealed significant differences between the two delay groups at baseline, *t*(24) = 2.84, *p* < 0.01, during acquisition block 1, *t*(24) = 2.65, *p* < 0.01, and during acquisition block 3, *t*(24) = 2.61, *p* < 0.01. Mann–Whitney U tests corroborated each of these comparisons (baseline: U = 28.5, *p* = 0.008, *r* = 0.64; acquisition block 1: U = 27.0, *p* = 0.006, *r* = 0.65; acquisition block 3: U = 21.5, *p* = 0.002, *r* = 0.72), indicating that the group differences were not artifacts of distributional skew. By the extinction block, the two groups no longer differed, *t*(24) = 1.12, *p* = 0.27 (Mann–Whitney U = 55.0, *p* = 0.22, *r* = 0.30), but the two groups reached this convergence by moving in opposite directions. The delay group declined from acquisition block 3 to extinction, consistent with a typical extinction response, as reported in prior work, whereas the no-delay group continued to rise. A Mann–Whitney test on the change in kicks from acquisition block 3 to extinction confirmed this differential pattern (U = 120.5, *p* = 0.02, *p* = −0.55).

Dichotomizing time out of the gahvora at a single cutoff reduces statistical power and treats infants tested at 15 and 90 min post-gahvora as equivalent. The primary analysis therefore modeled time out of the gahvora as a continuous covariate rather than as a dichotomous grouping variable. A negative binomial GEE modeling on-leg kicks as a function of block, time since gahvora removal (centered), and their interaction yielded a significant block × time interaction, Wald χ^2^(4) = 12.71, *p* = 0.013, with main effects of block χ^2^(4) = 51.77, *p* < 0.001, and time, χ^2^(1) = 8.24, *p* = 0.004. The effect of time out of gahvora on kicking was strongest at baseline and the first acquisition block (rate ratio per 10 additional minutes = 1.20, 95% CI [1.06, 1.36], *p* < 0.004, and rate ratio = 1.21, 95% CI [1.08, 1.36], *p* = 0.001, respectively), attenuated through acquisition block 3 (rate ratio = 1.09, *p* = 0.25), and absent at extinction (rate ratio = 1.04, *p* = 0.58). Spearman correlations between time out of the gahvora and kicking at each block showed the same pattern: ρ = 0.48, 0.55, 0.47, 0.51, and 0.20 at baseline through extinction. A Spearman correlation between time out of the gahvora and the within-infant change in kicking from acquisition block 3 to extinction was negative and significant (ρ = −0.40, *p* = 0.048), replicating the extinction-divergence finding without relying on a cutoff: Infants who had been out of the gahvora for less time showed the largest increases at extinction. Time out of the gahvora shapes baseline activity and the form of the extinction response but does not determine acquisition; the categorical delay-group comparisons show the same pattern.

For the untethered leg, there was a main effect of block, *F*(4, 96) = 3.18, *p* < 0.01, partial η^2^ = 0.12, mirroring the temporal pattern observed for the tethered leg. However, neither the main effect of delay group nor the interaction was significant for the untethered leg, indicating that baseline differences and their effects on acquisition were specific to the leg experiencing the contingency.

To determine whether recent release from the gahvora affected the expression of learning, we compared acquisition ratios across delay groups. We computed two ratios with baseline kicking as the denominator: an acquisition ratio (acquisition block 3 ÷ baseline), which indexes learning during the contingency phase, and a retention ratio (extinction block ÷ baseline), which indexes response level when reinforcement was removed. Two infants with zero kicks at baseline (both no-delay) were excluded from ratio analyses. Because ratios of counts are typically skewed and contained extreme values in this sample, we tested group differences with Mann–Whitney U tests. The two groups did not differ in the acquisition ratios (no-delay: *Mdn* = 3.62, IQR [1.65, 6.30]; delay: *Mdn* = 1.66, IQR [1.29, 2.85]; U = 85.0, *p* = 0.25, *r* = −0.29), indicating that both groups increased their kicking from baseline to the end of acquisition at comparable rates relative to their own baselines. By contrast, the no-delay group showed a substantially higher retention ratio than the delay group (no-delay: *Mdn* = 3.33, IQR [2.29, 8.67]; delay: *Mdn* = 1.37, IQR [1.00, 2.14]; U = 101.5, *p* = 0.031, *r* = −0.54). This difference reflects the divergent response to contingency removal documented above—a sustained response in the no-delay group versus a decline in the delay group—rather than a difference in acquisition. The proportion of infants meeting the learning criterion was also numerically higher in the no-delay group (9 of 13, 69.2%) than in the delay group (5 of 13, 38.5%), though this difference did not reach significance, χ^2^(1) = 2.48, *p* = 0.12. We note, however, that the criterion-based index is also affected by baseline kicks that affects the original acquisition ratio: Because the 1.5 threshold applies a fixed multiplicative standard to baselines that differ substantially between groups, infants with lower baselines more easily exceed the threshold than infants with higher baselines. We therefore relied on the Mann–Whitney and GEE analyses, rather than the criterion-based index, for our inferences about learning. Together, these findings indicate that time out of the gahvora influenced initial motor activity levels and shaped how infants responded when reinforcement was removed, but did not determine whether infants could detect and learn the contingency. Both groups acquired the learned association at comparable rates; they differed in the form of their extinction response.

### 3.4. Vocalizations During Learning

**Figure 5:**
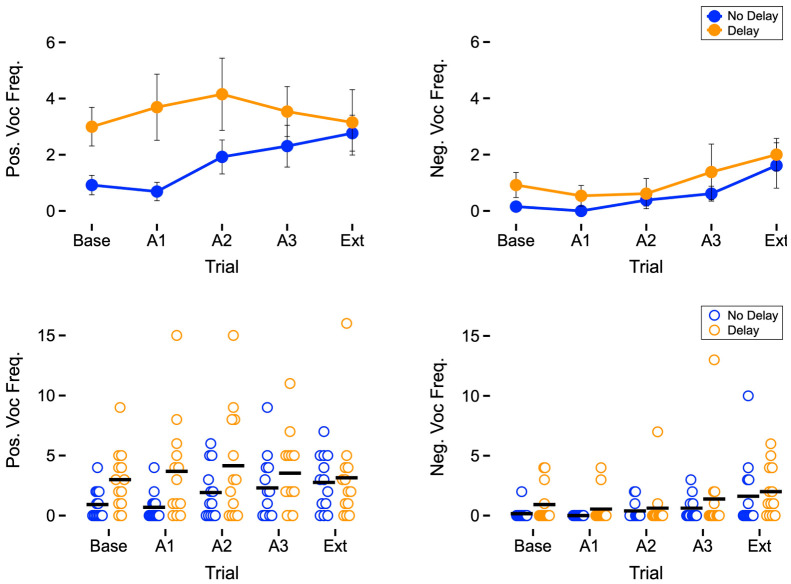
Frequency of positive and negative vocalizations across acquisition and extinction blocks for the no-delay (blue) and delay (orange) groups. The left panels show positive vocalization frequency, and the right panels show negative vocalization frequency. Top panels display group means with standard error bars; bottom panels display individual data points with group means indicated by horizontal lines. Positive vocalizations were present throughout acquisition for both groups. Negative vocalizations increased sharply during extinction (block 5), consistent with a frustration response following the violation of a learned expectancy.

**Figure 6:**
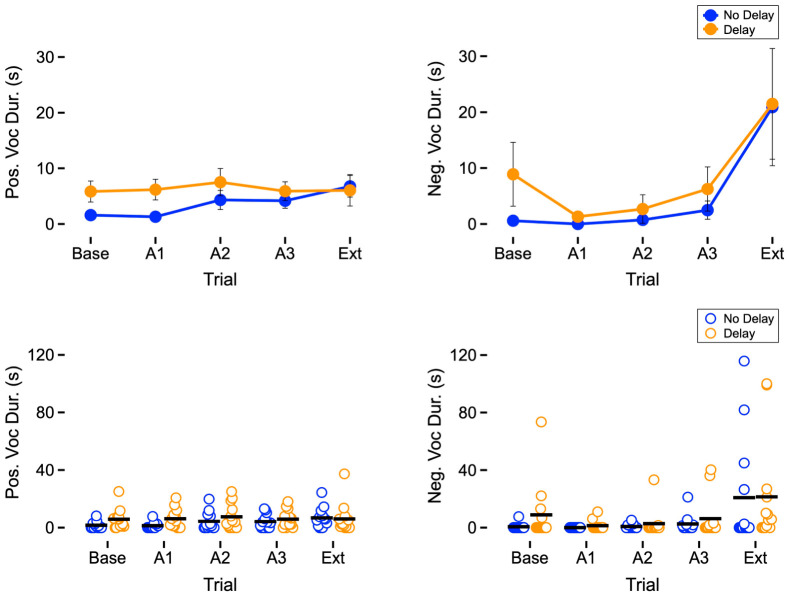
Duration of positive and negative vocalizations (in seconds) across acquisition and extinction blocks for the no-delay (blue) and delay (orange) groups. The left panels show positive vocalization duration, and the right panels show negative vocalization duration. Top panels display group means with standard error bars; bottom panels display individual data points with group means indicated by horizontal lines. Negative vocalization duration increased markedly during extinction (block 5), providing converging evidence of an extinction burst—a hallmark of contingency violation.

Beyond motor behavior, we examined whether infants’ affective expressions changed as they learned and then experienced removal of the contingency. Figure 5 shows the frequency of positive and negative vocalizations across trial blocks, and Figure 6 shows their duration.

A 2 (vocalization type: positive vs. negative) × 5 (block) mixed-design ANOVA on the frequency of vocalizations revealed a main effect of block, *F*(4, 100) = 7.16, *p* < 0.001, partial η^2^ = 0.22, and an interaction between vocalization type and block, *F*(4, 100) = 4.42, *p* < 0.01. As shown in Figure 5, infants vocalized more frequently as the test session progressed. During acquisition blocks 2 and 3, when infants were actively learning the contingency, they produced more positive/neutral vocalizations than negative ones. However, during the extinction block, when kicks no longer moved the mobile, infants showed a striking increase in negative vocalizations, suggesting that they detected the removal of the contingency and responded with frustration or distress.

Figure 6 shows that the duration of positive vocalizations remained relatively stable across blocks for both delay groups, but the duration of negative vocalizations increased dramatically during extinction (top right panel). This pattern held for both delay groups, indicating that the affective response to contingency removal was independent of initial activity levels.

Finally, we examined the possible relation between expressions of negative affect and kicking during the acquisition blocks. We found no relation between negative vocalizations during learning blocks and kicking, *t*(24) = 1.06, *p* > 0.05, confirming that infants are not excessively distressed during learning. Instead, they are changing the frequency of their kicking as they are learning the contingency between their movements and the mobile.

## 4. Discussion

We asked whether restrictive cradling practices in Tajikistan affect 3-month-old infants’ capacity to learn action–outcome contingencies—a fundamental building block of cognitive development. We used a well-established paradigm—mobile conjugate reinforcement—to test infants who spent most of their first 3 months bound in gahvora cradles day and night. Our findings advance existing knowledge in three important ways. First, infants reared under extensive movement restriction showed robust contingency learning when given the opportunity to act. Second, natural variation in time out of the gahvora—a within-culture comparison—allowed us to examine how recent motor experience shapes the expression of learning in real time. Third, affective responses to contingency detection and violation revealed that infants learn and form expectations about the consequences of their actions.

### 4.1. Contingency Learning Despite Restricted Movement

Despite spending many hours per day with limbs and body bound in gahvora cradles, Tajik infants demonstrated evidence of contingency learning. They increased kicking across the three successive acquisition blocks, differentiated the tethered leg from the untethered leg, and maintained elevated kicking during extinction. These patterns are the hallmarks of contingency detection in the mobile paradigm: Infants discovered that their kicks produced mobile movement, selectively increased activity in the operative leg, and retained the learned association even after the ribbon was disconnected. The leg differentiation is particularly telling. If the increase in kicking merely reflected a general “warming up” as infants became more active after release from the gahvora, both legs should have increased equally. Instead, the divergence between tethered and untethered legs emerged during acquisition and was most pronounced in the later acquisition blocks and during extinction—precisely the pattern expected if infants were encoding which specific action produced the contingent outcome.

The role of time out of the gahvora added nuance to these findings. We found no evidence that infants in the gahvora struggle against their bindings; they remain calm and relatively quiet in the cradle. Yet during the mobile learning task, infants expressed vigorous kicking and active engagement with the mobile. This basic observation suggested that infants can recognize changes in the environmental context that affords active responding, but they might need time to adapt. Indeed, infants who began the mobile task within minutes of gahvora removal started with lower baseline kicking than infants who had been free to move for a longer period beforehand, and the two groups showed different trajectories through the acquisition blocks. Yet by the extinction block, the groups converged—both showed comparable retention of the learned contingency. This pattern affirms that infants recently released from the gahvora needed time to adapt to their sudden freedom of movement before they could fully exploit the contingency, similar to rat pups fitted with unilateral limb weights that recalibrate their motor output before producing organized movement [21], or fetal rats that alter patterns of interlimb coordination when experiencing conjugate restriction of two limbs [21]. The adjustment period for the no-delay group did not prevent learning; it shifted its time course. This finding also argues against the possibility that the observed learning was simply a byproduct of increasing arousal. If infants were merely becoming more active over time, we would not expect the group-by-block interaction to be specific to the tethered leg—but it was.

The vocalization data provide a complementary window onto what infants understood about the contingency. During acquisition, when kicks reliably moved the mobile, infants produced predominantly positive or neutral vocalizations. During extinction, when the contingency was abruptly removed, negative vocalizations increased sharply. This pattern of extinction-induced frustration is a well-established marker of expectation violation in the mobile paradigm [40] and signals that infants formed a representation of the action–outcome relation. Negative affect during acquisition was not related to kicking rate, confirming that increases in kicking reflected learning rather than distress. The affective response to contingency removal was also comparable across delay groups, suggesting that when infants detected the contingency—regardless of how quickly they arrived at it—they formed equally strong expectations about its persistence. One alternative reading of the extinction-phase increase in negative affect is that infants were tiring or becoming frustrated toward the end of the session rather than detecting the contingency violation. Our data speak against this interpretation. The sharp rise in negative vocalizations occurred specifically in the extinction block, not as a gradual accumulation across acquisition blocks; a generalized fatigue account would predict a continuous increase, whereas the observed pattern was more step-like, at the point when the contingency was removed. Coders also monitored infants’ state throughout the session, and sessions were terminated if infants became distressed, reducing the likelihood that the extinction response reflects end-of-session exhaustion. A no-mobile control group—infants who experienced an equivalent session without contingent reinforcement—would be required to rule out time-on-task effects fully, and we acknowledge that comparison as a direction for future work.

### 4.2. What Restricted Movement Tells Us About the Foundations of Learning

Taken together, these findings speak to a broader question about what infants need from their early environments to develop basic learning capacities. The gahvora presents a naturally occurring case of motor restriction that is far more extensive than anything that could be ethically imposed in a laboratory. And yet infants reared in gahvoras learned the kicking-mobile contingency at rates qualitatively similar to infants from samples with relatively unrestricted movement histories. This outcome aligns with the idea that contingency detection is a robust and rapid capacity whose expression depends on current motor affordances rather than on accumulated movement experience. Infants did not need months of free kicking to discover that their legs could move a mobile—they figured it out within minutes. The mechanisms to support sensorimotor learning clearly are in place at this age, but how they are engaged and applied depends on environmental context. The foundations that permit motor learning doubtlessly are established much earlier, most likely during spontaneous movement in utero.

This finding has important implications for understanding why gahvora-reared infants show no long-term developmental lags despite the extent of their early restriction [19]. One possibility, suggested by the current data, is that the learning mechanisms underlying cognitive development do not depend on the quantity of self-generated movement during the first months of life. Rather, these mechanisms appear to be activated—and to function effectively—whenever infants are given the opportunity to act. The brief windows of freedom that Tajik infants experience daily when out of the gahvora for feeding, bathing, and social interaction may be sufficient to support the development of contingency sensitivity, provided those windows include responsive social partners and opportunities for self-generated action. In this sense, the quality of the out-of-cradle experience may matter more than its duration.

This interpretation helps clarify the contrast between gahvora-reared infants and orphanage-reared infants, who show significant and lasting delays across motor, cognitive, and linguistic domains [20]. On the surface, both populations experience restricted movement. But the contexts of restriction differ profoundly. Gahvora-reared infants are embedded in responsive caregiving environments—when they are out of the cradle, and often when they remain restricted within the cradle, caregivers attend to them, respond to their signals, and engage them socially. Orphanage-reared infants, by contrast, may lack movement opportunities and also contingent social interaction. If contingent experience—rather than movement per se—is the critical ingredient for early cognitive development, then gahvora-reared infants have it in concentrated form during their out-of-cradle periods, whereas orphanage-reared infants may lack it entirely. The current findings are consistent with this possibility, though they cannot directly test it.

Our data also contribute to a growing body of work that uses naturally occurring cultural variation as a window onto developmental processes [41,42]. Much of what we know about infant learning comes from Euro-American samples where infants have near-continuous freedom of movement, abundant objects, and attentive caregivers who structure play. By studying infants who develop under very different conditions, we can begin to distinguish which aspects of early experience are necessary for particular developmental outcomes and which reflect the specific affordances of a given cultural context. In prior work, we found that Tajik infants in their second year engaged with objects at rates comparable to U.S. infants despite having far fewer toys and spending a substantial amount of time restricted in the gahvora [43]. The present findings extend this pattern to the first months of life: even at 3 months, when gahvora use is at its peak, the capacity to learn from contingent action–outcome relations appears intact. Cultural childrearing practices shape the timing and context of developmental experience, but they do not necessarily constrain infants’ fundamental capacity to learn when opportunities arise.

Finally, these results speak to broader questions about the relation between motor development and cognitive development. Theories emphasizing embodied cognition propose that movement experience is not merely a context for learning but an element of it [5]. Our findings do not contradict this view but suggest refinement. The question may not be whether infants accumulate movement experience in general, but whether they can generate self-produced actions in the moment that yield contingent consequences. The no-delay infants—those tested almost immediately after release from the gahvora—showed that even a few minutes of adjusting to unrestricted movement was sufficient to support contingency detection. This suggests that the mechanism linking action to learning operates on a rapid timescale and does not require a long developmental history of free movement to be functional. What may be essential is the capacity for self-generated action itself, however recently acquired or re-acquired [2]. In other words, having some opportunities for high-quality contingency-response learning may be sufficient to promote typical motor development; having more opportunities may affect the timing of achieved “milestones” but not alter the course of developmental change.

### 4.3. Limitations and Future Directions

Several limitations should be noted. First, the duration of the 10 min cutoff used to distinguish delay from no-delay infants was selected *post hoc* after inspecting the distribution of time out of the gahvora. It was not derived from an *a priori* theoretical prediction. Second, the delay group reflects a procedural variable that the researchers observed rather than a manipulation, so the delay versus no-delay contrast is a *post hoc* subgroup comparison rather than an experimental contrast. Our sample was modest in size and drawn from a specific region of Tajikistan, limiting generalizability. The delay versus no-delay grouping, although informative, was based on naturally occurring variation in when infants happened to be removed from the gahvora relative to the researcher’s visit rather than on random assignment into delay groups. This means that unmeasured factors correlated with time out of the gahvora—such as infant state, hunger, or caregiver routine—could have contributed to group differences. Additionally, we did not include a comparison sample of unrestricted infants, either within Tajikistan or from another cultural context. Although our findings are qualitatively consistent with published reports of mobile conjugate reinforcement learning in Western samples, direct cross-cultural comparisons would strengthen claims about the equivalence of learning. We also assessed learning in a single session and did not test long-term retention; future work should examine whether gahvora-reared infants show the same memory consolidation over days or weeks that prior research documented in unrestricted samples [38]. Finally, although we measured negative affect and found that it did not account for learning, we did not collect comprehensive temperament assessments. Future studies could examine whether individual differences in temperament interact with movement restriction history to predict learning trajectories. Future work could also track whether the contingency learning observed here at 3 months relates to later cognitive and motor outcomes, and whether infants tested at ages when gahvora use begins to decline show different patterns.

## 5. Conclusions

Despite spending most of their early months of life with limbs bound in gahvora cradles, 3-month-old Tajik infants demonstrated robust contingency learning when given the opportunity to act on their environment. They detected that their kicks moved a mobile, selectively increased kicking of the operative leg, retained the learned association after the contingency was removed, and expressed frustration when their expectations were violated. The capacity for contingency detection looks resilient, emerging whenever infants have the opportunity for self-generated action—even after extended periods of restriction. Cultural childrearing practices shape the circumstances under which infants encounter contingent experiences, but they do not limit infants’ fundamental capacity to learn from them.

## Data Availability

The original data presented in the study are available on Databrary, https://databrary.org/volume/1425, accessed on 6 July 2026.
